# Extraction of total nucleic acids from bacterial isolates using the bioMérieux NucliSENS easyMAG total nucleic acid extractor

**DOI:** 10.1186/s12941-016-0168-7

**Published:** 2016-09-22

**Authors:** Eleanor A. Powell, Joel E. Mortensen

**Affiliations:** Diagnostic Infectious Disease Testing Laboratory, Cincinnati Children’s Hospital Laboratory, 3333 Burnet Avenue, MLC 1010, Cincinnati, OH 45229 USA

**Keywords:** DNA extraction, 16S rRNA sequencing, Bacterial sequencing

## Abstract

The BioMerieux NucliSENS easyMAG total nucleic acid extractor was evaluated for use on bacterial isolates in the clinical microbiology laboratory. Forty eight isolates were extracted, yielding quantifiable amounts of DNA for all isolates. The easyMAG is appropriate for DNA extraction from bacterial isolates and will be incorporated in the clinical laboratory.

The number of molecular methods and their importance in the clinical microbiology laboratory is rapidly increasing. Nucleic acid extraction from bacterial isolates is an important first step in several molecular test methodologies, including 16S rRNA sequencing for bacterial identification, whole genome sequencing, and other genotypic methods. While some clinical microbiology testing platforms have integrated nucleic acid extraction, others require separate nucleic acid extraction before testing may begin. Laboratory quality control has recently come under renewed scrutiny with the implementation of individual quality control plan (IQCP) requirements and as part of this, it is critically important to identify steps in a testing process that may result in testing failure or testing delays.

Inadequate nucleic acid extraction may result in an insufficient amount of nucleic acids, which can result in wasted reagents and delayed results. Manual extraction methods have traditionally been used, but these methods require extensive hands-on time and frequently have many steps, which increases the opportunity for method error or sample contamination [1]. The NucliSENS easyMAG total nucleic acid extractor (bioMérieux, Marcy l’Etoile, France) is a platform capable of total nucleic acid extraction from various sources, including blood, stool, and urine. The easyMAG extracts nucleic acids using magnetic silica beads. EasyMAG extraction of clinical samples such as respiratory samples, stool, and blood followed by molecular detection of viral and bacterial pathogens has been reported [2–4]. While the easyMAG has been used on a variety of clinical specimen types, the extractor does not include pre-set protocols for bacterial extraction. Although a small number of published studies have utilized the easyMag extractor to extract nucleic acids from bacterial isolates, these have typically been limited to a small range of species [5]. For the easyMAg to be utilized as the primary bacterial nucleic acid method in a clinical microbiology laboratory, the extractor must be capable of extracting nucleic acids from a wide variety of bacterial species. To determine the suitability of the easyMAG for routine bacterial nucleic acid extraction, the current study sought to examine the ability of the easyMag extractor to extract nucleic acids from a wide variety of bacterial isolates.

Forty-eight isolates from the American Type Culture Collection (ATCC) were selected to represent the wide variety of isolates typically encountered in a clinical microbiology laboratory (Table 1). Organisms were assigned to one of four groups: Gram-positive cocci, Gram-positive bacilli, Gram-negative bacilli in the family *Enterobacteriaceae*, and Gram-negative bacilli other than *Enterobacteriaceae*. A 1.0 McFarland suspension of each isolate in 0.45 % saline was made using the bioMérieux DensiCheck. 100 microlitre of each isolate was placed in the vessel portion of the easyMAG disposables and inserted in the instrument. DNA extraction was set up using the following parameters: program-specific 1.0.1, volume (input)-0.1 mL, elution volume-110 µL; lysis- on board. Ten microlitre of the resulting nucleic acid eluent was quantified using the Eppendorf Biophotometer (Eppendorf, Hauppauge, New York, USA).

**Table 1:** Bacterial species included in validation

Gram-positive cocci	Gram-positive bacilli	*Enterobacteriaceae*	Non-*Enterobacteriaceae* gram-negative organisms
*Staphylococcus epidermidis*	*Streptomyces albus*	*Escherichia coli* (3)	*Campylobacter jejuni*
*Staphylococcus aureus*	*Exiguobacterium mexicanum*	*Providencia stuartii*	*Campylobacter coli*
*Enterococcus faecium*	*Clostridium septicum*	*Salmonella enterica*	*Pseudomonas aeruginosa* (2)
*Streptococcus pyogenes*	*Actinomyces viscosus*	*Shigella sonnei*	*Prevotella melaninogenica*
*Staphylococcus saprophyticus*	*Propionibacterium acnes*	*Plesiomonas shigelloides*	*Stenotrophomonas maltophilia*
*Enterococcus faecalis*	*Corynebacterium striatum*	*Enterobacter cloacae*	*Alcaligenes faecalis*
*Streptococcus pneumoniae*	*Clostridium perfringens*	*Vibrio vulnificus*	*Acinetobacter baumannii*
*Streptococcus mitis*	*Actinomyces pyogenes*	*Klebsiella oxytoca*	*Haemophilus influenzae*
*Streptococcus agalactiae*	*Streptomyces griseus*	*Citrobacter freundii*	*Burkholderia cepacia*
	*Listeria monocytogenes*	*Proteus mirabilis* (2)	*Neisserisa meningitidis*
		*Klebsiella pneumoniae*	*Pseudomonas putrefaciens*
		*Serratia marcescens*	*Pasteurella multocida*
			*Bordetella bronchiseptica*
			*Moraxella catarrhalis*

For each of the four groups, the two isolates with the lowest nucleic acid concentrations were submitted for 16S rRNA sequencing. When these two isolates were from the same genus, the isolate with the next lowest nucleic acid concentration was submitted, ensuring more diverse representation of each group. Sequencing results were compared to the ATCC identification of the organism to ensure correct identification. Four additional isolates yielding higher extracted DNA concentrations (7.8–21.6 ng/µL) were also sequenced.

Extraction with the easyMAG resulted in detectable amounts of nucleic acids for all isolates (Table 2). Gram-positive organisms generally resulted in lower nucleic acid concentrations and A_260_/A_280_ ratios than Gram-negative organisms. A_260_/A_280_ is a measure of nucleic acid purity [6]. A_260_/A_280_ of 1.8 represents pure DNA, while an A260/A280 of 2.0 represents pure RNA. Lower values are typically the result of protein contamination. For those isolates with the lowest concentrations of nucleic acids in each group, 16S rRNA sequencing was successful. It is expected that isolates yielding higher concentrations of DNA would also sequence, as low DNA quantity is frequently cited as the reason isolates cannot be sequenced. To confirm this, four isolates with higher extracted DNA concentrations were also sequenced, and all yielded identifications. Low A_260_/A_280_ also did not affect downstream 16S rRNA testing, as isolates with A_260_/A_280_ as low as 1.05 were sequenced successfully. Low ratios are likely the result of protein in the DNA extract, which does not prevent downstream testing. It is expected that nucleic acids from easyMAG extractions will be suitable for other PCR-based methodologies.

**Table 2:** Concentration and A260/A280 of extracted total nucleic acid

	DNA concentration (ng/µL)		A_260_/A_280_	
	Median	Range	Median	Range
Gram-positive cocci	8.5	7.4–14.5	1.35	1.05–1.70
Gram-positive bacilli	9.1	8.0–37.8	1.47	1.09–1.80
*Enterobacteriaceae*	15.9	12.1–20.4	1.63	1.27–1.94
Non-*Enterobacteriaceae*	17.4	7.8–22.0	1.67	1.02–1.82

In-house nucleic acid extraction can decrease turn-around-time of downstream testing and minimizes the risks associated with sending potential pathogens to other laboratories. Coupling laboratory-performed nucleic acid extraction with nucleic acid quantification allows laboratories to perform additional manipulation on isolates that initially do not yield adequate amounts of nucleic acid. Identification of these isolates before downstream testing can prevent unsuccessful testing, save reagents and testing time, and decrease overall time to test result. While this was not required in the current study, lysis can be performed manually rather than on the instrument to extend lysis time and additional physical lysis steps such as grinding can also be added. Additional studies are necessary to develop protocols for nucleic acid extraction from acid-fast bacilli and fungi. The efficacy, flexibility, and ease-of-use of the bioMérieux NucliSENS easyMAG total nucleic acid extractor make it suitable for use in the routine clinical microbiology laboratory.
